# Cerebellar transcranial direct current stimulation in neurological disease

**DOI:** 10.1186/s40673-016-0054-2

**Published:** 2016-09-02

**Authors:** Roberta Ferrucci, Tommaso Bocci, Francesca Cortese, Fabiana Ruggiero, Alberto Priori

**Affiliations:** 1 Fondazione IRCCS Ca’ Granda, Ospedale Maggiore Policlinico, via F. Sforza 35, 20122 Milan, Italy; 2 Dipartimento di Scienze della Salute, Università degli Studi di Milano, via Rudinì 8, 20142 Milan, Italy; 3 III Clinica Neurologica, Polo Ospedaliero San Paolo, via Rudinì 8, 20142 Milan, Italy

**Keywords:** Ataxia, Cerebellar tDCS, Dystonia, Essential tremor, Parkinson’s disease

## Abstract

Several studies have highlighted the therapeutic potential of transcranial direct current stimulation (tDCS) in patients with neurological diseases, including dementia, epilepsy, post-stroke dysfunctions, movement disorders, and other pathological conditions.

Because of this technique’s ability to modify cerebellar excitability without significant side effects, cerebellar tDCS is a new, interesting, and powerful tool to induce plastic modifications in the cerebellum.

In this report, we review a number of interesting studies on the application of cerebellar tDCS for various neurological conditions (ataxia, Parkinson’s disease, dystonia, essential tremor) and the possible mechanism by which the stimulation acts on the cerebellum.

Study findings indicate that cerebellar tDCS is a promising therapeutic tool in treating several neurological disorders; however, this method’s efficacy appears to be limited, given the current data.

## Background

The cerebellum is an interesting and fascinating part of the central nervous system, which plays a fundamental role in movement execution and motor control in humans [1].

Beyond its role in balance and motor control, the human cerebellum has been extensively studied for its potential roles in learning, cognition, emotions, and behaviour [2, 3]. Indeed, experiments using neurotropic viruses as transneuronal tracers have demonstrated that the cerebellar output influences not only the primary motor cortex but also the premotor, prefrontal, and parietal areas [3].

The interaction between the cerebellum and the cerebral cortex is based on multiple closed-loop circuits, which have been traditionally considered anatomically and functionally distinct from the cerebro-basal ganglia loops. However, recent anatomical experiments have shown that the dentate nucleus projects disynaptically to the striatum [4] and that the subthalamic nucleus of the basal ganglia projects to the cerebellar cortex [5]. This interconnection between the cerebellum and the basal ganglia provides an explanation for the cerebellar involvement in disorders that are commonly associated with basal ganglia dysfunction (for example, Parkinson’s disease (PD) and dystonia) [6, 7].

Recent findings concerning the anatomical and functional connections between the cerebellum and the basal ganglia may help to characterize the role of the cerebellum in Parkinson’s disease [7]. The cerebellum was shown to receive a dopaminergic projection from the ventral tegmental area/substantia nigra pars compacta [8–10] and to exhibit dopamine D1–3 receptors [11, 12]. Moreover, pathological changes in the cerebellum following dopaminergic degeneration have been reported in patients with Parkinson’s disease and animal models [13, 14]. Functional neuroimaging studies demonstrated increased activation in the cerebellum in patients with PD during motor execution [15–20], during the motor learning process [21–23], as well as in the resting state [24]. A PET study on trial-and-error sequence learning determined that mildly affected patients with PD showed a similar performance with respect to controls but activated four times as much neural tissue, including the cerebellum [25]. It was suggested that the functional role of the increased activity or connectivity in the cerebello-thalamo-cortical loop in PD could be to compensate for hypofunction in the striato-thalamo-cortical circuit [7]. Indeed Yu and colleagues [20] observed a significant negative correlation between the blood oxygen level–dependent activation in the ipsilateral cerebellum and the contralateral putamen while performing a right hand pressing task.

There is strong evidence that the cerebellum also plays an important role in dystonia [6, 26–30]. Recent studies have shown subtle defects in cerebellar Purkinje cells in autopsy specimens from patients with cervical dystonia [31, 32]. Furthermore, imaging studies revealed that abnormalities in the basal ganglia frequently coexist with abnormalities in the cerebellum [33–37]. These data suggest that cerebellum function is incompletely characterized and is probably more expansive than expected.

Because of the multiple cerebellar connections and the broad variety of motor and non-motor functions, the field of cerebellar stimulation with non-invasive techniques (particularly transcranial magnetic stimulation (TMS) and transcranial direct current stimulation (tDCS)) is enjoying great success among scientists in the last several years. These techniques enable researchers to investigate neural networks non-invasively and enable them to obtain more information regarding cerebellar physiology and to promote neural plasticity [38].

In 1995, Ugawa [39] found that a single magnetic cerebellar pulse inhibited the amplitude of the motor evoked potential, evoked by TMS delivered a few milliseconds later over the contralateral motor cortex [39, 40]. This inhibition was mediated through the pathway between the cerebellum and the primary motor cortex and was termed cerebellar brain inhibition (CBI).

Controversial results were observed regarding the effects of tDCS on CBI. Galea and colleagues [41] found that anodal tDCS induced an increase of CBI, suggesting that it could act by stimulating tonic Purkinje cell activity, thereby potentiating the inhibitory effect of the dentato-thalamo-cortical pathway on the motor cortex. Nevertheless, Doeltgen and colleagues [42] found that anodal tDCS resulted in a reduction of CBI, suggesting that instead of Purkinje cells, anodal tDCS could modulate superficially located inhibitory interneurons projecting to them, or alternatively, cerebello-thalamo-cortical projections could target inhibitory interneurons within the primary motor cortex. Because of its ability to modify cerebellar excitability without significant side effects, cerebellar tDCS is a new, interesting, and powerful tool to induce plastic modifications in the cerebellum.

In this report, we review a number of interesting studies regarding the application of cerebellar tDCS for different neurological conditions (Table 1) and the possible mechanism by which the stimulation acts on the cerebellum. Cerebellar tDCS has been applied not only to patients affected by primary degenerative cerebellar disorders (e.g., cerebellar ataxias) but also to patients affected by disorders that primarily affect the basal ganglia (e.g., PD, dystonia). Therefore, by modulating cerebellar excitability, cerebellar tDCS could potentially represent a promising therapeutic strategy for movement disorders.

**Table 1:** Cerebellar Transcranial Direct Current Stimulation (tDCS) studies

Author & year	Sample	Trial type	Polarity and number of sessions	Stimulation electrode position	Reference electrode position	Current strength and duration	Outcome	Online/Offline procedure	Follow-up	Results
*Parkinson’s disease*										
Ferrucci et al. (2015) [47]	*N* = 9 (74.3 ± 8)	Randomized, double blind, cross-over	A/S 5 daily tDCS	Whole cerebellum or bilateral M1	Right deltoid muscle	2 mA, 20 min	UPDRS (III-IV), PDQ8, BDI, word recall, spatial cueing, SRTT	Offline	1–4 week	A tDCS over M1 and cerebellum improved: UPDRS(dyskinesia section) score by about 20 %.
*Dystonia*										
Sadnicka et al. (2014) [50]	*N* = 10 (not reported)	Randomized, double-blind	A/S single tDCS	Right cerebellar cortex	Right buccinator muscle	2 mA, 15 min	RMT, AMT, RC, CSP, VAS	Offline	No	Negative
Bradnam et al. (2015) [48]	*N* = 16 8 patients (59 ± 13) 8 healthy subjects (61.21 ± 11.73)	Randomized, double-blind	A/C/S single tDCS	Right cerebellar cortex	Right buccinator muscle	2 mA, 20 min	ADDS, WCRS, MEPs, MFS, APP	Offline	No	A tCDCS improved: APP by 12.81 %. A-C tDCS reduced handwriting MSF (A: 8.47 %; C: 9.6 %).
*Essential Tremor*										
Gironell et al. (2014) [51]	*N* = 10 (71.4 not reported)	Randomized, double blind, cross-over	C/S 5daily tDCS	Bilateral cerebellar cortex	Fp 1, Fp2	2 mA, 20 min	TCRS, accelometric recording, self-reporteddisability scale	Offline	4 weeks	Negative
*Cerebellar Ataxia*										
Grimaldi et al. (2013) [52]	*N* = 9 (mean age 51.3 ± 14)	Single blind, sham-controlled	A/S	Right cerebellar cortex, vermis	Contralateral supra-orbital area	1 mA, 20 min	SR, MCT, Computerized Posturography	Offline	No	A tCDCS reduced the amplitudes of long-latency stretch reflexes
Grimaldi et al. (2014) [53]	*N* = 2 (mean age 46 ± 4.24)	Single blind, sham-controlled	A/S	Right cerebellar cortex, Left M1	Contralateral supra-orbital area, right supra-orbital area	1 mA, 20 + 20 min	SARA, Upper limb tremor (postural and action tremor), dysmetria	Offline	No	A tCCDCS reduced: the PSD peak by 38.63 and 41.42 % in both patients, the magnitude of low frequency oscillations by 46.9 and 62.3 % respectively, and the onset latency of the hypermetria by about 41 and 45 %.
Benussi et al. (2015) [54]	*N* = 19 (mean age 53.8 ± 18.4)	Randomized, double blind, cross-over; sham-controlled	A/S	Cerebellar cortex	Right deltoid muscle	2 mA 20 min	SARA, ICARS, 9HPT, 8 MW	offline	No	A tCDCS improved: SARA by about 10 %, ICARS by 12 %, 9HPT by 11 %, 8 MW by 11 %.

*A* anodal tDCS, *ADDS* arm dystonia disability scale, *AMT* active motor threshold, *APP* average pen pressure, *BDI* beck depression inventory, *C* cathodal tDCS, *ICARS* International Cooperative Ataxia Rating Scale, *M1* motor cortex, *mA* milliampere, *MCT* Mechanical Counter Test, *Min* minutes, *MEPs* motor evoked potentials, *MSF* mean stroke frequency, *Offline* the subject receives stimulation before and after executing the task, *Online* the subject receives stimulation during the task, *PDQ-8* Parkinson’s disease questionnaire 8, *Fp* prefrontal areas, *PSD* power spectral density, *S* sham tDCS, *SARA* scale for the Assessment and Rating of Ataxia, *SR* Stretch reflexes, *SRTT* serial reaction time task, *tCDCS* transcranial cerebellar direct current stimulation, *tCCDCS* transcranial cerebello-cerebral direct current stimulation, *tDCS* transcranial direct current stimulation, *TCRS* tremor clinical rating scale, *UPDRS* Unified Parkinson’s disease rating scale, *VAS* visual analog scale, *WCRS* writer’s cramp rating scale, *9HPT* Nine-Hole Peg Test, *8MW* 8-Meter Walking Time

## Cerebellar tDCS studies

Evidence from clinical studies suggests that the cerebellum may be involved in the pathophysiology of movement disorders, such as dystonia [6], essential tremor [43], PD [44] and cerebellar ataxia [45], and may be a useful target for tDCS intervention [38, 46].

### Parkinson’s disease

Levodopa-induced dyskinesias (LIDs) most likely arise through the cerebello-thalamo-cortical circuit. To evaluate how cerebellar tDCS affects LIDs and cognitive function in PD, Ferrucci and colleagues [47] delivered bilateral anodal (cerebellar tDCS or M1-tDCS) and sham tDCS. After patients received anodal cerebellar tDCS and M1-tDCS for 5 days, the UPDRS IV score (dyskinesias section) improved; conversely, the other variables were unchanged after sham tDCS, cerebellar tDCS, and M1-tDCS.

Despite the small sample size, these preliminary results show that anodal tDCS applied for five consecutive days over the motor cortical areas and the cerebellum improves the LIDs of patients with PD. This finding provides the first evidence that cerebellar tDCS affects LIDs, thus corroborating and extending our current knowledge on the role of the cerebellum in the pathophysiology of PD [7]. Important limitations of this study are the lack of a pharmacological correlation analysis, no inclusion of healthy subjects, and the demonstration that administering L-dopa turns facilitation caused by anodal tDCS into inhibition and prolongs the inhibitory effects of cathodal tDCS.

### Dystonia

To investigate the role of the cerebellum in dystonia, Bradnam and colleagues [48] evaluated whether cerebellar tDCS improves handwriting and cyclic drawing kinematics by reducing CBI evoked by TMS in patients with writing dystonia. These researchers examined the kinematic measures, mean stroke frequency with handwriting and fast cyclic drawing, and average pen pressure during light cyclic drawing at baseline in patients and a control group. They examined these measurements after anodal, cathodal, and sham tDCS. The results showed that cerebellar anodal tDCS reduced handwriting mean stroke frequency and average pen pressure, and increased speed and reduced pen pressure during fast cyclic drawing. Kinematic measures were not associated with a decrease in CBI.

Primary dystonia is characterized neurophysiologically by reduced inhibitory mechanisms and an abnormal regulation of plasticity responses. The method commonly used to induce LTP-like (long-term potentiation) plasticity is paired associative stimulation (PAS). This protocol induces plastic changes in excitability in the human motor cortex [49]. In their study, Sadnicka and colleagues [50] used cerebellar tDCS to examine the electrophysiological parameters (resting and active motor threshold, motor cortex excitability, recruitment curves, and cortical silent period) in ten patients with writing dystonia. Patients completed a two-session study (sham and anodal) in which the cerebellum was stimulated along with simultaneous application of PAS.

Patients were also clinically evaluated with the writing movement subscore of the Writer’s Cramp Rating Scale and with a self reported visual analog scale.

The results were negative and do not provide evidence that anodal cerebellar tDCS is beneficial for patients after a single session.

Based on these limited and contradictory results, we conclude that the evidence supporting cerebellar tDCS, as a clinically viable method for treating dystonia is unconvincing. To the best of our knowledge, no one has investigated the effects of consecutive cerebellar tDCS sessions or the effects of combining it with motor training. Further research is needed to investigate ways for increasing the magnitude of the cerebellar tDCS effect.

### Essential tremor

Essential tremor (ET) is a common neurological disorder that is characterized by action tremor, which affects the upper limbs in at least 95 % of patients. Tremor is associated with a dysfunction in the basal ganglia and the cerebellar circuit, as well as several neurotransmitter systems projecting to both of these circuits. In a randomized, controlled and crossover study, Gironell and colleagues [51] evaluated the efficacy and safety of ten consecutive cerebellar tDCS sessions in patients with ET. Participants performed a tremor clinical rating scale (TCRS), a test that measures motor performance, daily living activities, or the patients’ subjective assessment, accelerometric recordings and a self-reported disability scale after receiving cathodal stimulation over both cerebellar hemispheres. The authors found no significant differences in the way tDCS affects outcome measures between baseline, day 10 and day 40 (30 days after the last tDCS session).

Although they investigated both the acute and long-lasting effects of tDCS, this small and preliminary study yielded negative results. It is not clear how the four tDCS electrodes were applied over the scalp. The authors have reported two cathodal electrodes placed symmetrically over both cerebellar hemispheres and two anodal electrodes over both prefrontal areas, without specifying if they were unilateral or cross montage. Moreover, it is questionable whether a discomfort questionnaire was administered along with the tDCS procedure and if the subjects were successfully blinded with 2 mA tDCS to the stimulation conditions. It would have been informative if the subjects had been asked if they received real or sham stimulation.

Given the ample evidence in the literature supporting the ability of cerebellar tDCS to enhance motor and non-motor function, the prospect of further trials for neurological disorders is encouraging.

### Cerebellar ataxia

Another recent study has investigated the role of tDCS to modulate the activity of the cerebellum in ataxic disorders and has reported interesting results.

To evaluate the effects of anodal tDCS on the cerebellum, Grimaldi and colleagues [52] conducted a study with nine ataxic patients. The authors studied the stretch reflexes (SR) in the upper limbs and examined upper limb dexterity and coordination using a mechanical counter test (MCT). These researchers also used a computerized posturograph during three experimental paradigms. Their results showed that anodal tDCS (1 mA, 20 min) over the right cerebellar hemisphere reduced the amplitude of the long latency SR (LLSR) response, but did not affect the short latency SR response or the MCT score compared to the baseline and sham group. Additionally, the postural parameters remained unchanged following sham or active stimulation of the region in front of the vermis.

These data collectively demonstrated that anodal tDCS applied over the cerebellum reinforces the inhibitory activity exerted by the cerebellar cortex over cerebellar nuclei. tDCS of the cerebellum appears to be a novel method to study the modulation of LLSR by the cerebellar cortex.

In a subsequent study, the same authors [53] performed tDCS over the cerebellum for 20 min immediately followed by tDCS applied over the contralateral motor cortex (20 min, 1 mA) (tCCDCS: transcranial cerebello-cerebral DC stimulation) to assess upper limb tremor and dysmetria in two patients with dominant spinocerebellar ataxia. They found that for the postural tremor, tCCDCS induced a reduction in the amplitude of the oscillations at the level of the index in both patients, as reported by quadratic power spectral density (PSD). tCCDCS also had a positive effect on action tremors, as observed by the drop in the magnitude of low-frequency oscillations from 62.3 to 46.9 % of the baseline values in patient 1 and 2, respectively. Remarkably, following tCCDCS, hypermetria occurred along with a reduction of the onset latency of the antagonist EMG activity in both patients.

Despite the small sample and the lack of follow-up precluding the investigators from assessing how long the effect lasted, these results are highly encouraging. The combination of tDCS of the cerebellum with tDCS of the motor/premotor cortex demonstrated that this technique may be considered a symptomatic therapeutic strategy for upper limb deficits in disabling cerebellar ataxia, representing a novel approach in the field of cerebellar neuromodulation.

An interesting therapeutic effect was also observed by Benussi and colleagues [54]. In a double-blind, randomized, sham-controlled study, they investigated whether a single session of cerebellar anodal transcranial direct current stimulation could improve symptoms in nine patients with ataxia. Each patient received both anodal and sham cerebellar tDCS stimulation (20 min, 2 mA) in two different sessions, separated by at least 1 week.

After cerebellar tDCS, there was a significant improvement in the functional clinical scores, as observed with the Scale for the Assessment and Rating of Ataxia (SARA), the International Cooperative Ataxia Rating Scale (ICARS), and in motor task measurement with the nine-hole peg test (9HPT) and 8-Meter Walking Time (8 MW) assessment within the entire cohort of patients. Specifically, the individual assessments of the four subscores on the ICARS scale revealed a significant improvement in the posture, gait and limb coordination subscores. Moreover, single-group analysis in the SCA (spinocerebellar ataxia) and the cerebellar variant of MSA (multiple system atrophy, MSA-C) cohorts demonstrated a significant effect from anodal cerebellar tDCS on SARA, ICARS, and 9HPT testing. Only in the SCA group was there a significant difference in the 8MW testing. There was no significant difference in the MSA-C group.

The investigators concluded that a single stimulation session applied to the cerebellar cortex can temporarily improve symptoms in patients with ataxia and might have therapeutic potential in these patients, but more powerful stimulation may be needed.

These preliminarily studies show that cerebellar tDCS is safe and can improve neurological symptoms, especially in patients with ataxia. While this finding is promising, the clinical efficacy of this technique in patients with neurological disorders remains to be definitively established by large, controlled clinical studies. Future research efforts should be directed toward identifying the optimal stimulation parameters (electrode montage and size, duration, intensity, number of sessions, on-line vs. off-line, duration of treatment), and potentially targeting specific types of neurological disorders or individual patients.

## Mechanisms of action

Two main questions concerning the mechanism of action of cerebellar tDCS are still unanswered: 1) whether cerebellar tDCS has polarity-specific effects or not and 2) whether it modulates cerebellar activity during or following a behavioural intervention (on-line *versus* off-line effects).

Differences in the reported effects are mainly due to the function explored, as different functions rely on different cerebellar areas with variable neural substrates and axonal orientation to the electrical field. Some authors have reported polarity-specific effects [41, 55–57], while others have not [58, 59]. This apparent discrepancy may be due to differences in the electrode size and the montage, as well as variations in the stimulation intensity [60]. In particular, while studies exploring cognitive and emotional domains have used a classical monopolar configuration, others focusing on motor functions have adopted a different montage, in which the return electrode is placed over the ipsilateral face. Only in the last study montage have polarity-specific effects been demonstrated [41].

Cerebellar tDCS probably has both on-line and off-line effects on cerebellar excitability. This finding would be in concordance with the effects elicited by tDCS in the cerebral cortex that are observable after both short-term and long-term delays and most likely interfering with long-term potentiation (LTP-like) phenomena [61]. From a cellular point of view, animal studies seem to suggest that the electrical stimulation of Purkinje cells mediates on-line effects [62], whereas depolarization of Golgi inhibitory neurons is responsible for long-lasting changes [63]. Purkinje cells represent the sole output from the cerebellar cortex, and their activation leads to the inhibition of the dentate nucleus, ultimately dampening motor cortex excitability, a phenomenon referred to as CBI [40]. Cerebellar tDCS may interfere with this connectivity, depending on its polarity and intensity. Nonetheless, electrical fields induced by cerebellar tDCS in humans are much smaller than those used in animals, thus making it difficult to compare their mechanisms of action [64, 65].

From a molecular perspective, cerebellar polarization may act on different neurotransmitters and signalling pathways [66]. For instance, the two main neurotransmitters in the cerebellum (glutamate and γ-amino-butyric acid) are both modulated when tDCS is applied over the sensorimotor areas [66]. In addition, the concentration of cerebellar myo-inositol is likely modified by weak electrical fields, as is the case for transcranial current stimulation [67]. Finally, cerebellar tDCS may interfere with dopaminergic transmission, probably through disynaptic pathways leading from the cerebellum to the intralaminar nuclei of the thalamus and to the dorsolateral putamen [4]. The cerebellum itself expresses all types of dopamine receptors that share similar properties with the striatal dopaminergic system [68]. The cerebellum also down-regulates the striatal D1 receptors through the deep nuclei and the thalamic relay [11].

As a whole, anodal and cathodal cerebellar tDCS likely exert effects through different, rather than simply specular, mechanisms of action on different cellular and molecular targets, in concordance with those reported both for the cerebral cortex [66] and spinal cord [56]. The polarity of cerebellar tDCS after-effects may also depend on the pre-existing excitability state, as well as with task-induced activity, as described for cortical stimulation [69]. Elucidation of the physiological variables underlying tDCS after-effects and evidence for the existence of a cerebellar homeostatic-like plasticity would be critical areas to investigate extensively in future studies.

## Conclusions

Cerebellar tDCS is a novel, safe and effective neurostimulation technique for non-invasive modulation of cerebellar excitability. Depending of the different pathophysiologies of human diseases, it has been successfully used for the treatment of movement disorders, ranging from cerebellar ataxia to dystonia, as well as for the control of levodopa-induced dyskinesias in Parkinson’s disease patients. Modelling studies have demonstrated that the cerebellum is the structure mainly affected by cerebellar tDCS, without a significant current spreading to the brainstem or the occipital cortex.

Studies on healthy subjects have demonstrated that cerebellar tDCS influences the pain experience, nociceptive perception [55, 56], and cognitive function [46].

However, further studies are needed to elucidate this technique’s mechanisms of action and areas of influence. Moreover, it is worth remembering that cerebellar involvement in non-motor (i.e., cognitive) functions remains to be systematically evaluated.

Despite the wide heterogeneity of the limited data available for review, we will try to offer some practical operative suggestions for those wishing to approach cerebellar tDCS to treat patients with neurological disease.

First, whereas anodal tDCS improves motor functions over the right cerebellum, cathodal cerebellar tDCS seems to be ineffective. The second suggestion concerns stimulation duration and intensity. The optimal repetition rate and duration to promote tDCS-induced plasticity remains undetermined.

A reasonable choice might be 2 mA for 20 min, using a common electrode size of 35 cm^2^ (generating charge densities ranging from 0.034 to 0.068 C/cm^2^) in repeated daily sessions (5 days). For instance, given that a recent study of major depression concluded that treatment should continue for several weeks or should combine stimulation of the cerebellum and cerebral cortex to achieve an optimal clinical response, the same might apply to neurologic patients. At present, because most of the available data on cerebellar tDCS-induced motor improvement concerns patients with ataxia, these patients appear to be the most likely to respond positively.

Further research efforts should aim to identify the optimal stimulation parameters (site, electrode montage and size, duration, intensity, number of sessions, online vs. offline, duration of treatment), potentially targeting specific types of neurological disease and individual patients.

Although current findings indicate that cerebellar tDCS may be a promising therapeutic tool for treating several neurological disorders, its efficacy appears limited and awaits further substantiation in large, randomised controlled clinical studies.
